# Restrictive Blood Transfusion Policy for the Management of Anemia in Palliative Care in Finland

**DOI:** 10.1089/pmr.2024.0050

**Published:** 2024-12-23

**Authors:** Reino Pöyhiä, Sari Hämäläinen, Karen Neoh, Annamarja Lamminmäki

**Affiliations:** ^1^ Department of Clinical Medicine, University of Eastern Finland, Kuopio, Finland.; ^2^ Department of Medicine, Kuopio University Hospital, Wellbeing Services County of North Savo, Kuopio, Finland.; ^3^ St Gemma’s Hospice, Leeds, United Kingdom.; ^4^ Departments of Oncology and Palliative Care, Kuopio University Hospital, University of Eastern Finland, Kuopio, Finland.

**Keywords:** anemia, end-of-life care, palliative care, transfusion

## Abstract

**Objectives:**

Restrictive blood transfusion policy has been shown to be effective in managing anemia. However, treatment of anemia in palliative (PC) and end-of-life (EOL) care remains understudied. The aim of this study was to examine Finnish PC physicians’ attitudes and practices in the management of anemia.

**Methods:**

A structured questionnaire asking clinicians about how they treat anemia in PC was developed with a focus on blood transfusion. In addition, a previously published collection of clinical case scenarios was included. Physician’s recall of their use of red blood cell (RBC) transfusions in 2021 was also asked. The questionnaire was first delivered at an annual meeting of the Finnish Association for Palliative Medicine in 2022 and subsequently, a Webpropol form was emailed to the members of the society.

**Results:**

A total of 94 (28%) doctors at an average age of 46.5 years responded. Of these 80% were specialists and 75% had a special competence in palliative medicine. RBC transfusions were given in less than 25% of patients, average hemoglobin was b ≤ 78 g/L. Transfusions were given for clinical symptoms such as fatigue, angina, and weakness without systematic measurement of symptom severity. Hematinic levels were investigated, but iron was given seldom. Clinical scenarios were answered similarly as previously in the UK. National guidelines for anemia in early PC or EOL care are lacking but would be deemed to be helpful by one-third of those who completed the questionnaire.

**Conclusion:**

Physicians apply the restrictive policy in blood transfusions for PC patients in Finland. Other treatments for anemia are not often used. Both prospective studies and national guidelines are needed.

## Key Message

Specific etiology of anemia is seldom investigated and treatments other than red blood cell (RBC) transfusions are not given in palliative care (PC) in Finland. The majority of Finnish PC physicians apply restrictive transfusion policy to their anemic patients triggered by a mean hemoglobin 78 g/L. Less than 25% of their patients have received RBC transfusions in PC.

## Introduction

Anemia is common among patients with advanced disease. The prevalence of anemia has been reported as 68% in women and 77% in men receiving palliative care (PC) when WHO criteria (hemoglobin, Hb <120 g/L in women and <130 g/L in men) were applied.^1^ In a retrospective analysis of hematologic values of 2416 patients in British PC services, the prevalence of moderate (Hb 80–110 g/L) and severe (Hb <80 g/L) anemia was 35% and 3%, respectively.^2^ Multiple factors contribute to anemia including stage of disease, bleeding, and vitamin deficiencies in PC patients.

Anemia is associated with poorer outcomes in patients with chronic diseases who are receiving active treatment.^3–7^ However, in PC, the significance of anemia is much more difficult to understand. First, the typical symptoms of anemia such as fatigue, tiredness, and dyspnea are often similar to symptoms of patients under PC services who are not anemic, regardless of the underlying disease.^8^ Second, those receiving PC may not benefit from life-prolonging treatments,^9,10^ and the treatments may also cause harm, particularly in the end-of-life (EOL) care.

Unfortunately, there is a lack of standardization in the approach to the management of anemia in PC.^11^ As an example, a trigger level of 70–90 g/L prior to red blood cell (RBC) transfusions^12–14^ is recommended but this is not based on randomized and controlled studies in PC. Furthermore, a systematic review of blood transfusion in adult PC concluded that RBC transfusion may provide symptom relief but for how long and by how much is still unknown.^15^

There is very little information available about how anemia is managed in PC patients in different countries. Studies from UK, Sweden, Turkey, and Nepal suggest variable attitudes and very liberal practices among physicians treating PC patients with anemia.^11,16–19^ According to these few reports, RBC infusions are still commonly used in dying patients but without comprehensive investigation of etiology and defined outcome measures. The aim of this study was to investigate, how Finnish physicians working in PC units treat anemia in their patients.

## Materials and Methods

No patient-identifiable data were collected; therefore, formal ethical approval was not required. A permit to run the study was obtained from the Ethics Committee of Kuopio University Hospital and the board of the Finnish Association for Palliative Medicine. The participation in the study was voluntary, which was also clearly stated at recruitment.

A questionnaire (see Supplementary Data S1) was developed in a specialist group of the authors, who all had special competence in palliative medicine and an extensive experience in PC. Prior to delivery, the questionnaire was evaluated by two other independent physicians. The questionnaire collected information about physicianś attitudes and practices in treating anemia in PC and more specifically in EOL care. In Finland, EOL care means holistic and multiprofessional PC during the last days and weeks before anticipated death.^20^ Details of the assessment of hemoglobin, hematinics (B12, folate), and iron metabolism in screening and detection of anemia were included in the questionnaire. RBC transfusions, monitoring side-effects of blood transfusions, and measurement of patient’s weight prior to transfusions were also documented. Furthermore, the responders were asked to state if national guidelines for RBC transfusions are needed. Doctors were asked to recall how they practiced in relation to RBC transfusions given during 2021.

Five clinical scenarios were presented to the physicians for judgment (Table 1). Four of them, published previously^21^ were included with permission of the author, and the answers were compared to those received from the UK survey. For all cases, the responders were asked to choose the best option for the management of anemia from the following: (a) active monitoring but not transfusion currently, (b) arrange transfusion of 1 RBC unit and monitor his/her response, (c) arrange transfusion 2 RBC units and monitor his/her response, (d) admit to hospital for further investigations and/or possible transfusion, and (e) other. Information on age, gender, Finnish medical specialty, training status, and whether they had achieved special competence in palliative medicine was also collected.

**Table 1.** Clinical Scenarios Presented in the Questionnaire

**Case 1.** Your patient is a 78-year-old man with metastatic prostate cancer, with bone metastases, who has progressed on hormone treatment and is not having any other active anticancer treatment. He also has a history of COPD. He has hemoglobin of around 101 g/L and over the last few months, this has fallen gradually to 85 g/L. He has no signs of active bleeding. He has been generally feeling less well including feeling fatigued and breathless, has a poor appetite, and has been losing weight, he does not have a low mood. He has a performance status of 3. You are seeing him as an outpatient.
**Case 2.** A community CNS (clinical nurse specialist) has seen a 70-year-old lady with gastric cancer, at oncology MDT, it was decided to treat her cancer with the best supportive care. She lives with her husband. Over the last week, she has felt more tired and has noticed her stools have become intermittently dark. Her hemoglobin has fallen from 101 g/L to 74 g/L over the last 2 weeks, and she needs more input from her husband, she is hemodynamically stable.
**Case 3.** A 62-year-old woman has been admitted to the hospice for the management of her complex pain due to bone metastases from breast cancer. She has been on NSAIDs and steroids and has developed hematemesis. She is currently hemodynamically stable and her hemoglobin has fallen from 101 g/L to 68 g/L. Her NSAID and steroids have been stopped.
**Case 4.** You are reviewing a 58-year-old man in an outpatient’s clinic. He has advanced colon cancer and is no longer under routine follow-up from oncology. He has come to see you as he is struggling with fatigue. He has no pain or nausea and has no signs of active bleeding. He has not noticed any change to his stools. His hemoglobin has been around 90 g/L and over a month has fallen to 75 g/L. He has generally been feeling less well and spending more time in bed with a performance status of 2. He is hemodynamically stable.
**Case 5.** A 78-year-old man with advanced COPD, diabetes, and end-stage ischemic heart disease (LVEF 20%). The patient is in palliative care. Hb 70 g/L. He suffers from dyspnea but has no pain. He can walk only a little inside but sits or lays on the bed for the most time.

CNS, central nervous system; COPD, chronic obstructive pulmonary disease; LVEF, left ventricular ejection fraction; MDT, multidisciplinary team; NSAID, nonsteroidal anti-inflammatory drug.

The questionnaire and clinical scenarios were distributed to the participants in the annual meeting of the Finnish Association for Palliative Care (FAPC) in 2022 and delivered thereafter to the members of the society using Webprol. The FAPC has 286 members with special competence in palliative medicine; 63% of them are geriatricians, general practitioner, or oncologists, and the rest of physicians with other specialties, 86% female physicians, and 83% under the age of 65.

### Statistical analysis

SPSS software was used for statistical analysis of data. Mean (with SD) values or percentages are given when appropriate. Correlation coefficients were calculated, and Chi-square test was used to detect statistically significant differences in responses between groups of responders with different educational backgrounds, work experience over or less than three years, and with different ages. A *p* < 0.05 was set as a statistically significant difference.

## Results

A total of 331 physicians working in PC received the questionnaire. Ninety-four (28%) doctors responded. Their demographic data are presented in Table 2. The majority of the specialist doctors were geriatricians or general practitioners (*n* = 49), while the rest of them (*n* = 25) came from a variety of medical specialties including anesthetics, acute medicine, gynecology, internal medicine, oncology, pediatrics, and respiratory medicine. Fifty-six (60%) responders had been working in units with less than 200 palliative patients annually and 31 (33%) in larger units.

**Table 2.** Demographics of the Responders

Variable	N/mean (SD)
F/M	82 (87%)/12 (13%)
Mean age (SD), year	46.5 (12.5)
Specialist/nonspecialist (*N*, %) in any medical discipline	74 (78%)/20 (22%)
PM competence/PM trainee/not training (*N*, %)	46 (49%)/23 (24%)/25 (27%)
Mean (SD) years since received PM competence	4.8 (4.3)
Work place: outpatient unit/hospice department/HAH/other department	34 (36%)/30 (32%)/35 (37%)/41 (44%)

A specialist physician means that the doctor has been approved as a specialist by the National Supervisory Authority for Welfare and Health, Valvira.

F, female; HAH, hospital at home; M, male; *N*, number of specialist or nonspecialist physicians and with special competence on palliative medicine (palliative competence or training in that such); PM, palliative medicine; SD, standard deviation.

### Laboratory tests relevant to anemia

The frequency of ordering a full blood count and various biochemical parameters to diagnose anemia was rarely applied in PC provided by our responders (Tables 3 and 4).

**Table 3.** How Often Do You Check Hb?

Frequency	*N* (%)
Never	1 (1)
Routinely all patients	19 (20)
If previously low Hb	49 (52)
Patient’s request	28 (30)
Family’s request	18 (19)
Always before red cell transfusion	62 (66)
After red cell transfusion	22 (23)
Other reasons (anemia-related symptoms, bleeding, acute change)	49 (52)

Numbers of respondents (with % of all respondents).

Hb, hemoglobin; *N*, number of responders.

**Table 4.** Checking Biochemical Parameters in Anemic Patients in Palliative Care

	Never	Seldom	Often	Always
S-folate	17 (18)	52 (55)	23 (24)	3 (3)
S-B12 vitamin	13 (14)	56 (60)	22 (23)	3 (3)
Other vitamins	38 (40)	26 (28)	2 (2)	1 (1)
S-Fe	27 (29)	42 (45)	10 (11)	3 (3)
S Ferritin	16 (17)	45 (48)	25 (27)	4 (4)
S-Transferrine	18 (19)	46 (49)	19 (20)	3 (3)
S-Transf saturation	18 (19)	47 (50)	18 (19)	3 (4)

The numbers refer to the number of responders (with % of all).

S, serum; Transf, transferrine.

### RBC transfusions

Indications for RBC transfusions in PC are based on clinical observations and listed in Table 5. Forty-seven (51%) doctors considered that RBC should not be given as part of EOL care. However, a significantly (*p* < 0.05) higher proportion (67%, *n* = 14) of physicians with more than three yearś experience in PC would sometimes give RBC transfusions to their patients at the EOL phase than less experienced doctors (36%, *n* = 4). The reasons for RBC transfusions in EOL care were similar to those for PC with one exception. Fourteen physicians reported they would give RBC transfusions to try and help a patient symptomatically before an important event or to try and remain at home for longer if a patient or a loved one requests. Most physicians (63/96; 66%) do not use any specific tool for the evaluation of the need for RBC transfusion, and only 30 would use Edmonton Symptom Assessment Scale. A total of 57 (61%) doctors do not measure patient’s weight prior to transfusion. The majority of responders discuss the transfusion with the patient (*n* = 87/93%) and with their loved one (*n* = 69/73%). When RBC transfusion is requested, 36 (38%) doctors use only one RBC unit and 36 two units. About 79% of the responders reported no observed transfusion-related complications, and the mean (SD) observed rate of transfusion-associated complications was 6.3 (5.7) % of patients. Most physicians (90%) monitor the effect of the transfusion and record this with narrative documentation in the patient notes. The practice of RBC transfusions in 2021 is reported in Table 6.

**Table 5.** Clinical Indicators and Measures for Red Cell Transfusions

Indication	*N* (%)
Increased weakness	73 (78)
Low Hb and angina	79 (84)
Dyspnea	77 (81)
Other (tiredness, malaise, bleeding, several symptoms simultaneously)	15 (16)
ESAS	30 (32)
WHO functional scale	8 (9)
QoL scale	2 (2)
No scale rutineously	63 (67)
Weight: no /always/sometimes	5761/1112/26 (28)

Numbers of responders (with % of all) are given.

ESAS, Edmonton Symptom Assessment Scale; QoL, quality of life; Hb, hemoglobin.

**Table 6.** Estimate of RBC Transfusions during 2021

Estimate % of transfused patients	*N* (%) of responders
0	4 (4)
<10	48 (51)
10–25	32 (34)
26–50	1 (1)
51–75	0
>75	0
NA	9 (10)

Numbers (*N*) and percentages of responders and percentage estimate for transfused patients of all patients are given.

NA, no answer.

A mean (SD) trigger value of Hb was 78 g/L (4.5) for RBC transfusions. Yet around half (52%) of doctors did not feel they could state a level of Hb to initiate transfusions. A total of 35 (37%) doctors wished and 34 (36%) did not desire national guidelines for RBC transfusions in PC, which are currently lacking in the country.

### Other treatments

A total of 13 (14%) and 15 (16%) of doctors would never prescribe oral or intravenous (iv) iron, respectively, to their patients. Sixty-one (64%) and 75 (78%) doctors administer oral or iv iron, respectively, to less than 25% of their patients. Forty-three (46%) doctors would give while 18 (19%) doctors would never give platelets to bleeding patients.

### Clinical case scenarios

The responses for clinical cases and their comparisons with previously published data^21^ are presented in Table 7. There were no substantial differences in reported practices between Finland and UK.

**Table 7.** Case Reports in Comparison with UK^21^

Case	Recommendation for care									
	1 RBC unit		2 RBC units		Follow-up		Hospitalization		Other	
	FIN	UK	FIN	UK	FIN	UK	FIN	UK	FIN	UK
1	25 (27)	57 (20)	6 (6)	59 (20)	46 (49)	137 (47)	5 (5)	7 (2)	10 (11)	29 (10)
2	18 (19)	31 (11)	45 (48)	113 (39)	4 (4)	19 (7)	18 (19)	61 (21)	4 (4)	65 (23)
3	4 (4)	22 (8)	48 (62)	101 (35)	2 (2)	18 (6)	35 (38)	111 (38)	3 (3)	37 (13)
4	24 (26)	66 (23)	35 (37)	130 (45)	14 (15)	45 (16)	5 (5)	7 (2)	13 (14)	41 (14)
5	29 (31)	NA	22 (24)	NA	27 (29)	NA	1 (1)	NA	12 (13)	NA

Figures represent absolute numbers (%) of the respondents. The total numbers of respondents were 93 in Finland (FIN) and 293 in the United Kingdom (UK).

RBC, red blood cell.

## Discussion

The main finding of our study is that the PC clinicians investigate and treat anemia on the basis of patients’ symptoms, rather than routinely for every patient. However, symptom checklists or quality-of-life instruments are not frequently used in the evaluation. The levels of hematinics are very seldom checked, nor are these corrected if abnormal. RBC infusions were used for less than 25% of patients on average, but there was wide variation in reported practice. Half of our responders would not give RBC infusions as part of EOL care at all.

An average trigger value of Hb prior to RBC was 78 g/L, although 52% of the doctors could not state any exact value indicating the necessity of blood transfusion. These observations are in line with those reported from UK,^14^ and the average trigger value indicates that Finnish physicians follow a restrictive policy of RBC infusions in PC units. A recent systematic review^22^ including 48 studies with 21,433 patients, though mostly not in PC, has provided evidence for the safety of restrictive transfusion policy. The international recommendations for PC are based on expert opinions and recommend a pretransfusion Hb at the level of 70–90 g/L.^23^

It is unclear whether RBC transfusions are associated with deterioration of clinical condition, or whether some patients are given RBC transfusions when they are close to the end of their life and therefore have a short prognosis following this. A systematic review found that 23% to 35% of patients with advanced cancer died within two weeks after their transfusions.^24^ Adverse effects from RBC transfusions are rare but may affect those near the end of their lives more commonly; for example, they are likely to have several risk factors for developing transfusion-associated circulatory overload (TACO). TACO has been described as the most common and often underdiagnosed cause of transfusion-related death. The risk of TACO is increased in patients with cancer, compromised health state, elevated pro-inflammatory cytokine levels, older age, and chronic kidney and liver failure.^25^ The current data about RBC transfusions remain insufficient to inform the safety of transfusion policies in PC.

Although anemia was associated with depression, poor quality of life, and reduced well-being in a retrospective study of PC patients with advanced heart disease,^26^ there are no randomized trials to show the effect of treating anemia on these symptoms. Few observational studies have reported some amelioration of symptoms after RBC transfusions for short duration, but in the absence of control groups, the effect cannot be fully evaluated.^15^ Yet still the treatment, and particularly, RBC transfusions are given by our responders for symptom control, in keeping with previous studies.^21^ As an example, a higher trigger Hb has been proposed for patients with angina pectoris,^27,28^ but so far, angina pectoris has not been shown to worsen or occur in anemic patients significantly more frequently than in other individuals.^26^ In PC, cardiac pain can be well alleviated with nitrate medications and opioids.^29^ Previous studies have suggested that transfusions of RBC may also worsen the outcome of coronary artery disease.^30,31^

Regarding the practice of giving RBC transfusions, many physicians in our study would give 1 or 2 units of RBCs. Yet the weight of the patients is rarely measured which is important in the assessment for risk of TACO. The transfusion-associated complication rate was very low (6.3%), which is in the same range (3%–6%) as reported previously.^15^ However, because our participants do not systematically monitor their patients before and after RBC transfusion, this estimate may not be accurate. Currently, national guidelines are lacking in Finland about PC RBC infusions, which a third of our responders would desire.

Other treatments of anemia, such as B12, folate, and iron were rarely chosen by Finnish doctors in PC. Similar findings were reported in UK too.^21^ This again is not surprising, because there is a lack of controlled studies of treating vitamin or iron deficiency in palliative. Yet iron might be an effective intervention because it has been found to improve the well-being of anemic patients in PC.^32^ A recent systematic review has indicated that restrictive transfusion policy is associated with better outcomes than liberal use even in bleeding patients.^33^

In this study, we compared the Finnish physicians’ responses to those from UK and Ireland using similar clinical vignettes. The doctors in both countries responded very similarly. Interestingly, all doctors seem to prefer 2 units of blood over 1 unit for transfusion, although patients’ weight is seldom measured before transfusion. Also, because of anemia, hospitalization was seldom considered to be needed. In the current study, we included a new case scenario without cancer but the answers to this case were not considerably different from those to other cases.

In Finland, all physicians with special competence in palliative medicine have received similar training which aligns with the recommendations of European Association for Palliative Care.^34^ Additionally, the national definitions of palliative and EOL care have been adopted from European standards.^10^ Multiprofessional PC is provided within public care for all patients when needed and according to the national guidelines.^20^ Although the response rate was only 28%, the demographics of our responders are very similar to those of the members of the FAPC, the vast majority of its members being female doctors. Also, the average age and variety medical specialties of our responders were close to the general profile of the society’s members. So, we believe that we have been able to give a representative picture of national PC practices in Finland.

The limitation of the current study is that we were not able to explore actual patient data but only the attitudes of physicians. Also, the practices of the doctors were based on their retrospective memory. We did not ask them specifically about the use of specific treatments (B12/folate/erythropoietin) for anemia. However, since the responders very seldom check B12 and folate levels, we hypothesize they are not used regularly to treat anemia in Finnish PC units. Additionally, erythropoietin is warranted only for hematology or severe bleeding but not in PC in our country. Furthermore, we did not inquire about the attitudes on ethical issues, which also would be important when RBC transfusions are used for dying patients. It is likely that most people who donate blood and its components do not anticipate it being used in patients with a short prognosis of less than a few weeks.

In summary, the majority of physicians likely apply restrictive transfusion policy to their anemic patients and less than 25% of patients receive RBC transfusions in PC. However, etiology of anemia is seldom investigated, and national guidelines would be needed for using RBC transfusions in Finland. Prospective, controlled, and randomized studies should be conducted about the treatment of anemia in EOL care.

## Abbreviations Used

EOL: end-of-life
FAPC: Finnish Association for Palliative Care
Hb: hemoglobin
PC: palliative care
RBC: red blood cell
SD: standard deviation
TACO: transfusion-associated circulatory overload
