# Supplementary material for: Restrictive Blood Transfusion Policy for the Management of Anemia in Palliative Care in Finland
Source: Palliat Med Rep. 2024 Dec 23;5(1):578–84. doi: 10.1089/pmr.2024.0050 (PMC11848061; doi:10.1089/pmr.2024.0050)
Supplement: Supplementary Data S1 [file pmr.2024.0050_supp_datas1.pdf]

ANEMIAN HOITO PALLIATIIVISEN HOIDON POTILAILLA – kysely. HUOM! kysymykset koskevat VAIN pall hoidon potilaita

TREATMENT OF ANAEMIA FOR PALLIATIVE CARE PATIENTS– survey. NOTE! All questions are related ONLY to palliative care patients.

Laita rasti (X) sopivaan vaihtoehtoon tai kirjoita vastaus. PS = punasolu.

Choose the item suitable to you with (X); or write a correct answer. PS = red blood cell.

Ikä (Age): ____ v(yrs), Sukupuoli (Gender) : __ Nainen (Female) ___ Mies (Male) ____ En halua sanoa (I don´t want to say).

___ erikoislääkäri (specialist physician), mikä erikoisala (which medical specialty): ___________________, ____ erikoistumassa (in training to specialist)

Palliatiivisen lääketieteen erityispätevyys (Special competence of palliative medicine:

__ ei (no), __ koulutuksessa (in training), ___ kyllä (yes), suoritusvuosi (year of graduation): ________

1. Missä työskentelet (voit valita useampia). Where do you currently work (you may choose several options).

__ vuodeosasto (in-ward): ___ erikoissairaanhoito (special health care) ___ perusterveydenhuolto (primary health care)

__ avovastaanotto (pkl) (outpatient service)

__ kotihoito (home-care)

__ muu, mikä (other place, which): _________________

2. Vaikuttaako (palliatiivisen) potilaan sairaus PS-siirtojen tarpeellisuuteen. Does the diagnosis the patient has influence whether you give a RBC-transfusion.

__ ei (no)

__ kyllä, missä sairauksissa olet eniten määrännyt PS-siirtoja anemian hoitoon? Mainitse enintään 3 sairautta (yes, in which diseases have you prescribed RBC transfusions most often? Give 3 diseases at the maximum): _____________________-

__ en osaa sanoa (I can´t say)

3. Tutkitko potilailtasi Hb/Hkr (voit valita useita vaihtoehtoja) Do you check your patients´ Hb/Hcr (you may choose several options)

__ en koskaan (never)

__ rutiinisti kaikilta (routinely all patients)

__ jos aikaisemmin matala Hb (when Hb has been low previously)

__ jos potilas pyytää (when the patient requests)

__ jos omainen pyytää (when a loved one requests)

__ aina ennen PS siirtoa (always before I prescribe RBC transfusion)

__ PS siirron jälkeen (after RBC transfusion)

__ muulloin, milloin? (in other circumstances, when)? ____

4. Miten toteutat hematologisia sairauksia (verisyövät, myelodysplasia) sairastavien palliatiivisten potilaiden PS-siirrot? (How do manage RBC transfusions to patients with haematological diseases (haematological malignancies, myelodysplastic syndrome)?

___ samalla tavalla kuin muidenkin (in the same way as the other patients)

___ hematologin ohjeiden mukaisesti (according to the haematologist´s instructions)

___ en osaa sanoa/en hoida tällaisia potilaita (I can´t say/ I don´t have patients with haematological diseases

5. Voiko saattohoidossa antaa PS-siirtoja? Are RBC transfusions given in end-of-life (hospice) care?

__ Ei koskaan (never)

__ Kyllä, minkälaisessa tilanteessa? (Yes, in which situations?) ______________________________________

__ en osaa sanoa (I can´t say)

6. Selvitätkö aneemisilta potilailta, jotka ovat palliatiivisessa hoidossa vitamiini- ja rauta-arvoja? Merkitse rasti (x) sopiviin sarakkeisiin. Do you request the following tests for patients under palliative care. Indicate the most suitable option with (x)

**Tutkimus. Test**	**En koskaan Never**	**Harvoin Seldom**	**Usein Ofte**	**Aina Always**
S-folaatti/ S-folate				
S-B12				
Muu vitamiinipitoisuus: mikä/mitkä Other vitamin levels				
S-Fe				
S-ferritiini/S-ferritin				
S-transferriini/S-transferrine				
fP-Trfesat, transferriinin saturaatioaste/fP-transferr saturation				

7. Määräätkö potilaillesi per os tai iv Fe-kuureja. Sijoita rasti (x) sopivimpaan vaihtoehtoon. Do you prescribe Iron orally or iv to your palliative patients?

	En koskaan Never	< 25 % potilaista/patients	25-50 % potilaista	51-75 % potilaista	> 75 % potilaista
per os rauta (oral)					
iv rauta (iv)					

8. Ovatko trombosyyttisiirrot tarpeen palliatiivisilla potilailla? Are platelet transfusions indicated in palliative care?

___ Ei. No

___ Kyllä, milloin?Yes, when? ____

9. Mikä Hb arvo mielestäsi indisoi palliatiivisille potilaille PS siirron? Which Hb level should indicate RBC transfusion to a palliative care patients?

_____ g /l

_____ en osaa sanoa/I can´t say

_____ ei ole mahdollista asettaa tällaista Hb arvoa/ it is not possible to give a specific Hb level

10. Käytätkö kliinisiä indikaattoreita PS-siirtoihin? Jos käytät, mitä? Do you administer RBC transfusions on the basis of symptoms? Which?

__ en koskaan/never

__ lisääntynyt heikkous/increased fatigue

__ matala Hb ja rintakivut/Low Hb and angina

__ hengenahdistus/dyspnoea

__ muu: mikä? (other symptoms, which?)_______________________________________

11. Käytätkö ao mittareita arvioidessasi PS-siirron tarvetta? Do you estimate the needs of RBC transfusions using the following measures?

__ ESAS; __ WHO -suorituskykyluokka; __ 15 D tai muu elämänlaatumittari (other QoL measure)

__ en käytä mitään systemaattista indikaattoria (I don´t use these measures systematically); __ en osaa sanoa (I can´t say)

12. Mittaatko potilaan painon ennen PS siirtoa? Do you weigh the patient before RBC transfusion __ en (no) ___ kyllä (yes) ___ joskus (sometimes)

13. Keskusteletko PS-siirroista. Do you discuss decisions about RBC transfusions with (Several options can be chosen).

__ potilaan (patient) kanssa, __ omaisten (loved ones) kanssa, __ hoitajien (nurses) kanssa?

14. Miten monta PS-yksikköä määräät palliatiiviselle potilaalle? How many RBC units do you give per transfusion episode?

__ aina vain 1 yks (always only 1); __ vähintään 2 yks (at least 2); __ ”tarpeen mukaan” jopa > 2 yksikköä (”according to the needs of patient, even > 2 units)

15. Miten seuraat PS siirron vaikutusta: How do you assess the outcome (effect) of RBC transfusion?

__ ESAS ennen ja jälkeen (ESAS before and after) __ elämänlaatu-mittari ennen ja jälkeen (mikä?) QoL measure before and after ___________=

__ kuvaan sairauskertomukseen vasteen (narrative description) __ ei systemaattista dokumentointia (I don´t document the effect systematically)

16. Oletko havainnut palliatiivisilla potilailla merkittäviä komplikaatioita, jotka olisivat keskeyttäneet PS siirron tai aiheuttaneet muita ongelmia? Have you noticed significant complications, which led to stopping the RBC transfusion or caused other problems= ____ en (no), ____ kyllä (yes): n © ____ %:lla potilaista (% of the patients receiving RBC transfusions)

17. Arvioi, miten monta palliatiivista potilasta yksikössäsi hoidettiin 1.1-31.12.2021? Please, estimate how many palliative patients were in your unit 1.1.-31.12.2021.

__ < 50 ___ 51-100 ___ 101-200, __ 201-500, ____ > 500

18. Miten moni heistä potilas sai PS siirtoja? How many of these patients received RBC transfusions?

___ 0 ___ < 10 % ____ 10-25 % ____ 26-50 % ___ 51-75 %, > 75 %

19. Miten monta yksikköä siirrossa per potilas keskimäärin annettiin? How many units did a patient get in average per transfusion) ___ 1, ___ 2, ___ ≥ 3

20. Tarvitaanko mielestäsi veripalvelun yksityiskohtaisia ohjeita palliatiivisen potilaan PS- siirtoihin? Do you think that national guidelines would be needed for RBC transfusions for palliative patients?

__ Ei (No)__ Kyllä (yes) __ En osaa sanoa (I can´t say)

Potilastapaukset. Clinical case scenarios (translated in Finnish) used previously in UK survey.

Valitse kullekin potilaalle seuraavista ao vaihtoehdoista paras rastittamalla (X): A, Hb arvoja ja yleisvointia seurataan, ei näillä tiedoilla PS siirtoja; B: 1 PS yksikön siirto ja vasteen seuranta; C: 2 PS yksikön siirto ja vasteen seuranta; D: Potilas otetaan sairaalaan lisätutkimuksiin ja varaudutaan PS siirtoihin; E: Muu, mikä? Jos valitset ”E:n”, kuvaa se vapaalla tekstillä lyhyesti.

Potilas	A	B	C	D	E: mikä?
78 vuotias mies, jolla prostata ca on levinnyt luustoon. Syövän aktiivihoito lopetettu, palliatiivinen hoitolinja. Lisäksi potilaalla on COPD. Hb taso on ollut 100 g/l luokkaa, mutta parin viime kuukauden aikana se on laskenut ad 85 g/l. Ei merkkejä verenvuodosta. Hänen yleisvointinsa on heikentynyt, hengenahdistus on lisääntynyt, ruokahalu on heikentynyt ja paino on laskenut, mutta mieliala ei pysynyt hyvänä. WHO:n suorituskykyluokka on 3. Hän on pkl-seurannassa vastaanotollasi.					
70 v kotihoidon potilas, jolla on mahasyövän takia supportiivinen hoitolinja, on käynyt väsyneemmäksi ja huomannut ulosteensa muuttuneen ajoittain tummiksi. Hb on laskenut 101 ad 74 g/l 2 viime viikon aikana. Hän asuu miehensä kanssa, jonka avun tarve lisääntynyt. Verenpaine- ja syketasot normaaleja.					
62 v nainen, jolla on luustoon levinnyt rintasyöpä, on otettu sairaalaan kipujen takia. Kipuja on kotona hoidettu NSAID-valmisteella ja kortikosteroidilla.					

Sairaalassa hän alkaa oksentaa verta. Hemodynamiikka on stabiili, mutta Hb on laskenut 101 ad 68 g/l. NSAID ja kortikosteroidi lääkitykset lopetetaan.					
Polikliinisena potilaana hoidat 58 v miestä, jonka levinneen colon-ca:n hoitolinja on palliatiivinen. Hän tulee vastaanotolle lisääntyneen heikkouden (fatigue) takia. Ei kipuja eikä verenvuotoja. Ulosteet ennallaan. Hb on laskenut viimeisen kuukauden kuluessa 90 ad 73 g/l. Yleiskunto on heikentynyt siinä määrin, että potilas on suurimman osan ajasta vuoteessa. WHO luokka 2. Ei hemodynaamisia ongelmia.					
78 v mies, jolla pitkälle edennyt COPD, diabetes ja loppuvaiheen iskeeminen sydämen vajaatoiminta (LVEF n 20 %). Palliatiivinen hoitolinja. Hb 70 g/l. Hengenahdistusta mutta ei kipuja. Kävelee vain vähän sisällä, etupäässä istuu tai makoilee.					

Kiitos!! Thanks!
